# Therapeutic Effect of *Amomum villosum* on Inflammatory Bowel Disease in Rats

**DOI:** 10.3389/fphar.2018.00639

**Published:** 2018-06-20

**Authors:** Zhu Chen, Wanye Ni, Caixia Yang, Ting Zhang, Shanhong Lu, Ronghua Zhao, Xiaojian Mao, Jie Yu

**Affiliations:** College of Pharmaceutical Science, Yunnan University of Traditional Chinese Medicine, Kunming, China

**Keywords:** *Amomum villosum*, immunoregulation, inflammatory bowel disease, intestinal microecology, T lymphocyte

## Abstract

**Introduction:** *Amomum villosum* Lour., a herbaceous plant in the ginger family, has been proven to be effective in treating gastrointestinal diseases. It has been listed in the Chinese Pharmacopeia as a legal source of Amomi Fructus. In our previous study, we demonstrated that treatment with extracts of *A. villosum* prevented the development and progression of intestinal mucositis. In the current study, we aimed to verify and explain the potential beneficial effects of *A. villosum* on inflammatory bowel disease (IBD).

**Methods:** The effect of water extracts (WEAV) and volatile oil of *A. villosum* (VOAV) were evaluated on the immunological role of T lymphocytes and intestinal microecology in IBD rats induced with 2,4,6-trinitrobenzenesulfonic acid (TNBS). Body weight, food intake, colon length/weight, and disease activity index (DAI) as well as tissue damage scores were evaluated. The inflammatory response to IBD was assessed by measuring the expression of myeloperoxidase, interleukin (IL)-17 (IL-17), interferon-γ (IFN-γ), IL-10, tumor necrosis factor-α (TNF-α), and transforming growth factor-β (TGF-β). The percentage of regulatory CD4^+^ T cells in rat spleen was measured by flow cytometry and effects on the microbial community were evaluated by 16S rDNA gene sequencing.

**Results:** All TNBS-induced rats showed typical clinical manifestations of IBD. IBD rats in the WEAV and VOAV treatment groups were effective in relieving body weight and appetite loss. Middle and high dosage of VOAV and WEAV significantly reduced the DAI, and tissue damage scores, whereas colon weight/length ratio was increase. All rats in the WEAV and VOAV groups showed significantly decreased IFN-γ levels and increased levels of IL-10 and TGF-β. Moreover, we observed that the percentage of regulatory CD4^+^ T cells was significantly enhanced during treatment with WEAV. In addition, administration of WEAV and VOAV effectively inhibited the release of enterogenic endotoxin, increased short-chain fatty acid-producing bacteria belonging to Firmicutes and Bacteroidetes, and decreased the abundance of Proteobacteria.

**Conclusion:** Treatment with WEAV and VOAV significantly attenuated intestinal inflammation in IBD rats, which was possibly associated with its regulation on inflammatory cytokine and CD4^+^CD25^+^FOXP3^+^ T cells. Moreover, WEAV and VOAV may help maintaining the balance of intestinal microecology.

## Introduction

Inflammatory bowel disease, including Crohn’s disease (CD) and ulcerative colitis (UC), is a chronic, relapsing and non-specific inflammatory disease with an unknown etiology (Li, 2014). Typical clinical manifestations of IBD include abdominal pain, diarrhea, and bloody mucopurulent stool. In China, the incidence and prevalence of IBD has significantly increased in recent years, which is probably due to an industrialized and westernized lifestyle, such as a high fat diet and an increase in intake of sugars, cigarette smoking, mental stress, antibiotics usage, and an imbalance of the intestinal flora (Ye et al., 2013, 2015). First-line treatments for IBD involve the use of immunosuppressants, corticosteroids, and anti-tumor necrosis factor (TNF) antibodies. Although these conventional methods may to some extent alleviate clinical symptoms, drug adverse reactions and high therapy-associated costs have been widely reported (Shafran et al., 2015). In recent years, herbal therapies, including Huangqin-Tang, aloe vera gel, extract of *Serpylli herba*, *Artemisia absinthium*, *Tripterygium wilfordii*, and *Andrographis paniculata* have shown beneficial effects in IBD patients (Ng et al., 2013).

Etiological studies have demonstrated that an aberrant immune response, genetic factors, and aberrations in the epithelial barrier and gut microbiota play important roles in the pathogenesis of IBD (Yoshiyuki et al., 2015). Gut microbiota play a crucial role in triggering, maintaining, and exacerbating IBD (Fedorak and Madsen, 2004). IBD is linked to a lack of physiological tolerance of the mucosal immune system to resident gut microbiota and pathogens (Pagliari et al., 2017). A reduction in bacterial diversity as well as a greater bacterial instability have been observed in patients with IBD. Increasing evidence has shown that overexpression of potentially proinflammatory microbes were exhibited in 2,4,6-trinitrobenzenesulfonic acid (TNBS)-induced IBD rats. Moreover, it has been extensively reported that a decreased abundance of Firmicutes and Bacteroidetes, and an increased abundance of Proteobacteria were related to the severity of IBD (Frank et al., 2007; Peterson et al., 2008). In addition, *Clostridium* clusters XIVa and IV consisting of *Clostridium*, *Ruminococcus*, *Lachnospira*, *Roseburia*, *Coprococcus*, *Eubacterium*, as well as *Anaerofilum* genera, and *Faecalibacterium prausnitzii* (*F. prausnitzii*), which belong to phylum Firmicutes, are reduced in IBD patients (Hiroko and Nobuhiko, 2017).

Intestinal immune system disorder is regarded as another main pathogenesis during the progression of IBD. In IBD rats, an imbalance between CD4^+^ T helper (Th) cells and regulatory CD4^+^ T (Treg) cells, which may further damage the pro/anti-inflammatory cytokine balance, has been reported (Zou et al., 2015). Therefore, immune-related inflammatory cytokines and Tregs are frequently-used targets for the treatment of IBD (Yang et al., 2014; Hang and Liu, 2015).

*Amomum villosum* Lour. (Zingiberaceae, a legal source plant of Amomi Fructus) is a classic traditional Chinese herbal plant (Commission of Chinese Pharmacopoeia, 2015). Previous pharmacological studies have shown that Amomi Fructus has great anti-ulceration, anti-diarrhea, anti-inflammatory, and antimicrobial activities (Huang et al., 2014). In our previous study, we demonstrated that *A. villosum* prevented the development and progression of intestinal mucositis after chemotherapy (Zhang et al., 2017). In this previous study, we found that *A. villosum* significantly alleviated endoenteritis by downregulating p38 MAPK and caspase-3 expression, strengthened the intestinal mucosal barrier, reduced the number of pathogenic bacteria, and increased the abundance of probiotics. Although *A. villosum* has been widely used in the treatment of gastrointestinal diseases, whether *A. villosum* can be applied in the treatment of IBD and its possible mechanisms of action involved are still lacking. In this study, we explored the effects of water extract and volatile oil of *A. villosum* on CD4^+^ T cells, immune homeostasis, and gut microbiota balance in a TNBS-induced colitis rat model.

## Materials and Methods

### Preparation of Water Extract and Volatile Oil From *A. villosum*

Fruit from *A. villosum* Lour. was collected in the Jingping County, Honghe Prefecture of Yunnan Province, China. Plants were identified as *A. villosum* Lour. by Jie Yu, an Associate Professor at Yunnan University of Traditional Chinese Medicine. Voucher specimens were deposited in the Herbarium of Pharmacognosy, Yunnan University of Traditional Chinese Medicine.

*A. villosum* fruit (400 g) was minced to powder and soaked in 3 L of water for 30 min at room temperature, then decocted three times with 3, 2, and 1 L of water, for 30 min each, respectively. Extracts were combined, condensed, and lyophilized. The water extraction yield of *A. villosum* (WEAV) was 14% of the crude drug.

Another 400 g of powdered sample of *A. villosum* was soaked in 3 L water for 30 min at room temperature. Volatile oil was extracted by steam distillation for 5 h, then dried with anhydrous sodium sulfate. The volatile oil yield of *A. villosum*(VOAV) was 3.6% of the crude drug.

### Animals

Adult male Sprague-Dawley rats weighing between 200 and 250 g were purchased from Da Shuo Biotech Co., Ltd., China (Certificate number SCXK 2013-24, Chengdu, China). Rats were kept on standard rat chow and water *ad libitum*, and were adaptively acclimatized for 7 days, with a 12 h light/dark cycle, a temperature of 23°C, and a 60% humidity. Experiments were performed in accordance with the guidelines of the Institutional Ethical Committee on Animal Care and Experimentations of Yunnan University of Traditional Chinese Medicine (R-0620160016). Surgeries were performed using sodium pentobarbital anesthesia and all efforts were made to minimize suffering of the rats.

### Induction of IBD and Treatment

Rats were fasted for 48 h with free access to water and anesthetized with 3% sodium pentobarbital (50 mg/kg). Next, colitis was induced according to the methods described by Randhawa et al. (2014). In brief, a lavage needle was inserted into the anus of each rat and the tip of the needle was advanced to 8 cm proximal to the anus verge. Next, a 5% TNBS solution (100 mg/kg) (Xiya Reagent, Chengdu, China) was dissolved in 0.25 mL 50% ethanol, and was injected into the colon. Rats in the control group were injected with 0.9% saline. After injection, all rats were kept in a head-down position for 1 min to avoid leakage of TNBS solution, then laid flat until consciousness was regained.

A total of 90 rats were randomly divided into nine groups (10 rats per group), including a control (CON) group; model (MOD) group; low-, medium-, and high-dose of WEAV (WEAV.L, WEAV.M, and WEAV.H) groups; low-, medium-, and high-dose of VOAV (VOAV.L, VOAV.M, and VOAV.H) groups; mesalazin enteric-coated tablets (MES) group (Sunflower Pharmaceutical Group Jiamusi Luling Pharmaceutical Co., Ltd., Haerbin, China). Rats in the CON and MOD group were gavaged with water. Rats in the WEAV.L, WEAV.M, and WEAV.H groups were orally administered with 200, 400, and 800 mg/kg dose of WEAV, respectively. Moreover, rats in the VOAV.L, VOAV.M, and VOAV.H groups were orally administered with 7, 14, and 28 mg/kg VOAV, respectively. Rats in the MES group were orally administered with mesalazin at 360 mg/kg. The concentrations of VOAV and WEAV were chosen from human clinical dosages of *A.villosum*, which were recommended by Commission of Chinese Pharmacopoeia (2015). The middle dose for humans was calculated as 4.5 g/kg in this study, and the rats dosages used were converted from the human dosage listed according to the body surface area. In addition, the yield of WEAV and VOAV were also considered. Treatments were started 6 h after IBD induction and continued for 9 consecutive days. Food intake and body weight were recorded daily.

### Disease Activity Index

Disease Activity Index (DAI) was measured according to a previously described method with some modifications (Murthy et al., 1993). Scores were given based on the percentage of weight loss (0, none; 1, 1–5%; 2, 5–10%; 3, 10–15%; and 4, >15%), stool consistency (0, normal; 1, pasty stool that does not stick to the anus; 2, pasty stool that does not stick to the anus; 3, pasty stool that stuck to the anus; and 4, watery stool), and rectal bleeding (0, hemoccult (-); 1, hemoccult (±); 2, hemoccult (+); 3, hemoccult (++); and 4, obvious blood in stool).

### Assessment of Colonic Damage

Rats were anesthetized with 3% sodium pentobarbital on day 9. The entire colon was removed and the length was measured. Then, colons were cut longitudinally along the mesentery, washed with ice-cold saline, and weighed. Macroscopic damage was scored according the method described by Bell et al. (1995) with modifications as follows: 0, no ulcers and inflammation; 1, mucosal hyperemia and edema; 2, ulceration without mucosal hyperemia and edema; 3, single ulceration and mucosal inflammation; 4, more ulceration and mucosal inflammation; and 5, severe ulceration extending >2 cm along colon length, mucosal hyperemia and edema.

Of each group, three representative samples were selected, fixed in 10% formalin and stained with hematoxylin and eosin (HE, submittal for inspection). The histological scores were assessed according to a method described by Neurath et al. (1995). Scores were as follows: 0, no colonic tissue damage; 1, low level of inflammatory cell infiltration, intestinal villus arranged neatly; 2, medium level of inflammatory cell infiltration, crypt damage, bowel wall thickening without invasion of the muscular layer; 3, high level of inflammatory cell infiltration, vascular proliferation, bowel wall thickening with an invaded muscular layer; 4, inflammatory cell infiltration, loss of goblet cells, vascular proliferation, and bowel wall thickening with an invaded muscular layer.

### Assessment of Th1-, Th2-, Th17-, and Treg-Related Cytokine Expression in Blood Samples and Colonic Tissue

From all rats, blood and colonic tissue samples were collected at the end of the experiment. The blood sample for assessment of Th1-, Th2-, Th17-, and Treg-related cytokine expressions were collected from rat retrobulbar venous plexus. Colonic tissue (100 mg) was homogenized in 1 mL 1× PBS and stored overnight at -20°C. Two freeze–thaw cycles were performed to break the cell membranes. Next, samples were centrifuged for 10 min at 10,000 rpm at 4°C to obtain serum and colonic tissue supernatants. Serum levels of IL-17, TGF-β, and IL-10 were determined using ELISA kits (Cusabio Biotech Co., Ltd., Wuhan, China). Levels of MPO, IFN-γ, and TNF-α in colonic tissue supernatants were analyzed by ELISA kits (Cusabio Biotech Co., Ltd., Wuhan, China). All assays were carried out using a Spectra Max PLUS 384 enzyme microplate reader (Molecular Devices Corporation, United States).

### Flow Cytometry Measurement of CD4^+^CD25^+^Foxp3^+^ Treg Cells

For analysis of Treg cells, rat spleens were grinded and washed with RPMI 1640 medium (Sigma, Germany) to obtain a single cell suspension. Then, cells were incubated with fluorescein isothiocyanate (FITC)-labeled anti-CD4 and phycoerythrin (PE)-labeled anti-CD25 antibodies for 25 min at 37°C. Cells were fixed and permeabilized with Fix/Perm solution. Cells were then washed in PBS and intracellular staining was performed using an allophycocyanin (APC)-labeled anti-Foxp3 antibody. Cells (1 × 10^6^) were resuspended in 500 μL flow cytometry staining buffer and analyzed using a FACSCalibur (Becton, Dickinson, Company, United States). Antibodies and the buffer used for analysis of Treg cells were purchased from eBioscience (San Diego, CA, United States).

### Assessment of Portal Vein Endotoxin Levels

Hepatic portal vein blood samples were collected from sodium pentobarbital anesthetized rats using disposable vacuum blood collection tubes. Blood samples were centrifuged for 10 min at 4000 rpm and 4°C. Lipopolysaccharide (LPS) concentrations in plasma were determined using a tachypleus amebocyte lysate test purchased from Chinese Horseshoe Crab Reagent Manufactory, Co., Ltd., Xiamen, China.

### Amplification and Sequencing of the 16S rDNA Gene

From the rats in each group, stool samples were collected using a 1.5 mL of sterilized cryogenic vials and stored at -80°C. From each group, three stool samples (100 mg each) were randomly chosen, weighed, and DNA was extracted using a stool mini kit (Omega Bio-tek, Norcross, GA, United States). The colony diversity in the colon, and the overall structural changes in gut microbiota were subjected to pyrosequencing of V4 regions of 16S rDNA, and amplified by PCR amplification using a 515F-806R primer set.

### Statistical Analysis

Statistical analysis was performed using SPSS version 19 software. Non-parametric data were analyzed by Wilcoxon rank sum test and multiple comparison for *post-hoc* test for significant pairwise differences. Parametric data were analyzed using one-way analysis of variance (ANOVA) with pairwise comparison as *post-hoc* test. *P* < 0.05 were considered significant. The Pearson’s correlation was conducted by SPSS statistics 19 software and graphics were prepared using Origin 6.1 software.

## Results

### General Physiological Features

**FIGURE 1:**
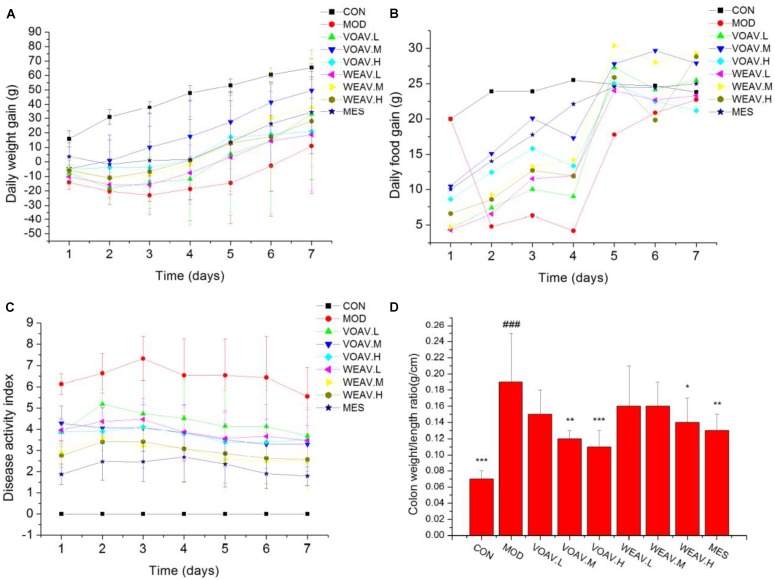
Effects of *A. villosum* administration on general physiological features in TNBS-induced rats. **(A)** Gain of daily weight; **(B)** gain of daily food; **(C)** disease activity index; **(D)** colon weight/length ratio. Rats were induced with TNBS (100 mg/kg) in a 50% ethanol solution. Low, middle, and high doses of water extract (WEAV) and volatile oil of *A. villosum* (VOAV) were orally administered. On day 9 of treatment, rats were anesthetized and colons were harvested. Data are expressed as the mean ± SD (*n* = 10). The symbol “^∗^” indicates a significant difference compared with the model group, and “#” indicates a significant difference compared with the control group. ^∗^*p* < 0.05, ^∗∗^*p* < 0.01, ^∗∗∗^*p* < 0.001, and ^###^*p* < 0.001.

General physiological features of rats, including body weight, food intake, DAI scores, colon weight and colon length were documented. Rats in the MOD group showed significant loss in weight and appetite when compared with rats in the CON group (*p* < 0.001). IBD rats also displayed higher DAI scores that were associated with higher incidences of diarrhea, rectal bleeding, and increased colon weight/length ratio. VOAV.M and VOAV.H significantly prevented weight and appetite loss in IBD rats (*p* < 0.05 or *p* < 0.001) (**Figures 1A,B**). Compared with rats in the MOD group, the DAI scores of rats in all treatment groups were significantly decreased (**Figure 1C**). In addition, treatment with VOAV.M, VOAV.H, and WEAV.H was effective in reducing colon weight/length ratio (**Figure 1D**).

### Macroscopic and Microscopic Assessment of the Colon

**FIGURE 2:**
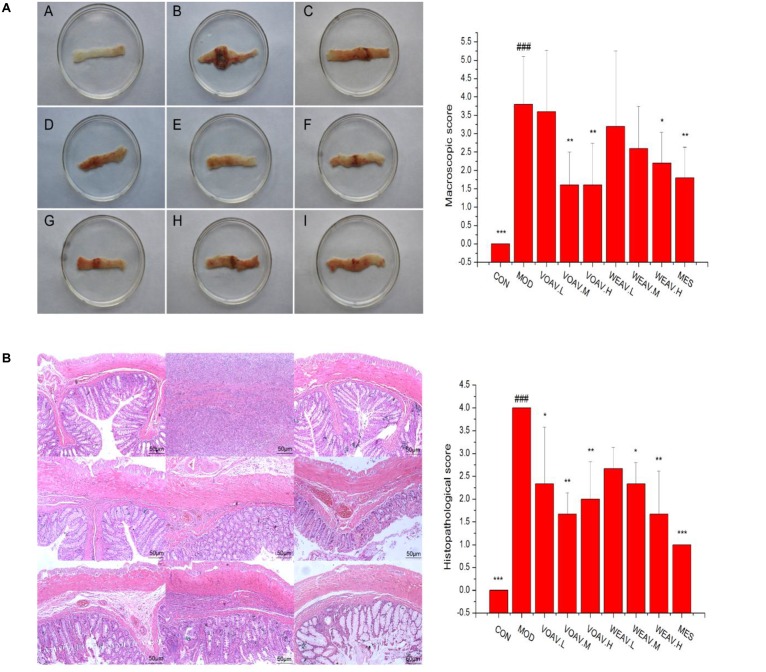
Effects of *A. villosum* administration on macroscopic and histopathological changes in TNBS-induced rats. **(A)** Macroscopic appearance of colonic tissue (*n* = 10); **(B)** histopathological appearance of colonic tissue (*n* = 3, magnification ×100). Rats were induced by TNBS (100 mg/kg) in 50% ethanol solution. Various doses of VOAV and WEAV were orally administered. On the 9th day, rats were anesthetized and the colons were excised, fixed in 10% formalin and stained with hematoxylin and eosin. (A) Colon of CON group; (B) colon of MOD group; (C) colon of VOAV.H group; (D) colon of VOAV.M group; (E) colon of VOAV.L group; (F) colon of WEAV.H group; (G), colon of WEAV.M group; (H), colon of WEAV.L group; (I), colon of MES group. Data are expressed as the mean ± SD (*n* = 10 or *n* = 3). The symbol “^∗^” indicates a significant difference compared with the model group, and “#” indicates a significant difference compared with the control group. ^∗^*p* < 0.05, ^∗∗^*p* < 0.01, ^∗∗∗^*p* < 0.001, and ^###^*p* < 0.001.

Macroscopic and microscopic scores of rats in the MOD group were significantly higher when compared to rats in the CON group (*p* < 0.001). As shown in **Figure 2A**, ulceration with mucosal hyperemia and edema was observed in rats in the MOD group. Such changes were markedly improved by treatment with VOAV.H, VOAV.M, WEAV.H, or MES (*p* < 0.01). Histological scoring revealed that all doses of VOAV, WEAV.M, and WEAV.H used in this study were effective in mitigating the severity of tissue lesions as characterized by the low level of inflammatory cell infiltration. Moreover, the intestinal villus arranged neatly and the number of goblet cells increased (*p* < 0.01 or *p* < 0.05, **Figure 2B**).

### Th1-, Th2-, Th17-, and Treg-Related Cytokine Expression

**FIGURE 3:**
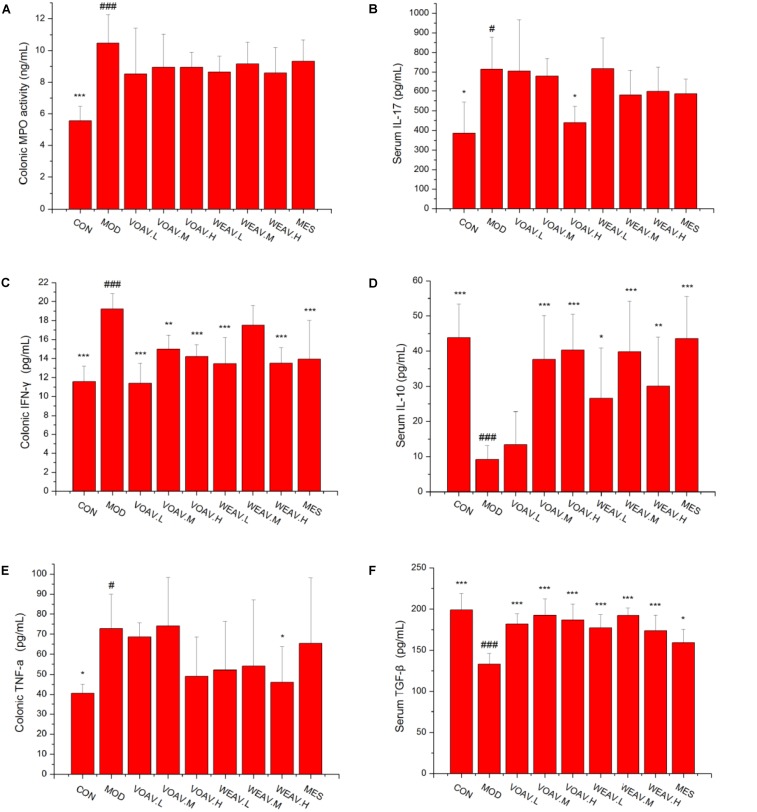
Effects of *A. villosum* administration on the expression of Th1-, Th2-, Th17-, and Treg-related cytokines in colonic tissue or serum of rats with IBD. **(A)** Colonic myeloperoxidase (MPO) activity; **(B)** serum Th17 signature cytokine IL-17; **(C)** colonic Th1 signature cytokine IFN-γ; **(D)** serum Th2 and Treg-related cytokine IL-10; **(E)** colonic Th1 signature cytokine TNF-α; **(F)** serum Treg-related cytokine TGF-β. Rats were induced by TNBS (100 mg/kg) in 50% ethanol solution. Various doses of VOAV and WEAV were orally administered. On the 9th day, blood and colonic tissue samples of all rats were collected and determined by ELISA. Data are expressed as the mean ± SD (*n* = 6). The symbol “^∗^” indicates a significant difference compared with the model group, and “#” indicates a significant difference compared with the control group. ^∗^*p* < 0.05, ^∗∗^*p* < 0.01, ^∗∗∗^*p* < 0.001, and ^#^*p* < 0.05, ^###^*p* < 0.001.

ELISA assays were performed to evaluate the levels of MPO, Th17 signature cytokine IL-17, Th1 signature cytokine IFN-γ, and TNF-α, Th2, Treg-related cytokine IL-10, and TGF-β. In general, MPO activity is a measure of neutrophil infiltration, and treatment with both VOAV or WEAV had no significant effect on MPO activity when compared to rats in the MOD group (**Figure 3A**). Levels of pro-inflammatory cytokines IL-17, IFN-γ, and TNF-α were markedly increased in rats in the MOD group. Treatment with VOAV.H and WEAV.H resulted in a clearly reduction of IL-17 and TNF-α levels (*p* < 0.05) by 41.6 and 36.8%, respectively (**Figures 3B,E**). Low and high-doses of VOAV and WEAV were effective in reducing IFN-γ levels (*p* < 0.001, **Figure 3C**). When compared with rats in the MOD group, expression of anti-inflammatory cytokine IL-10 was significantly enhanced by 77.5, 76.3, and 77.5% in VOAV.H, VOAV.M, and WEAV.H groups, respectively (**Figure 3D**). Moreover, the level of anti-inflammatory cytokine TGF-β of WEAV and VOAV treatments was significantly higher compared to that of rats in the MOD group (*p* < 0.001) (**Figure 3F**). These data suggested that treatment with VOAV and WEAV not only inhibited Th17 and Th1 responses, but also promoted Th2 and Treg responses in IBD.

### The Percentage of Regulatory CD4^+^ T Cells

**FIGURE 4:**
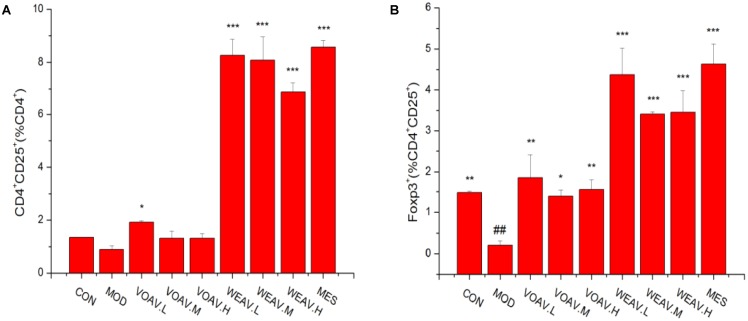
Effects of *A. villosum* administration on the percentage of CD4^+^CD25^+^Foxp3^+^ Treg cells. **(A)** Percentages of CD4^+^CD25^+^/CD4^+^ Treg cells; **(B)** Percentages of Foxp3^+^/CD4^+^CD25^+^ Treg cells. Rats were induced by TNBS (100 mg/kg) in 50% ethanol solution. Various doses of VOAV and WEAV were orally administered. On the 9th day, the Treg cells distribution in rat spleen were determined by flow cytometry. Data are expressed as the mean ± SD (*n* = 3). The symbol “^∗^” indicates a significant difference compared with the model group, and “#” indicates a significant difference compared with the control group. ^∗^*p* < 0.05, ^∗∗^*p* < 0.01, ^∗∗∗^*p* < 0.001, and ^##^*p* < 0.01.

Foxp3^+^ is a key cellular transcriptional factor, which allows differentiation of naive T cells into Treg cells. In this study, the percentage of CD4^+^CD25^+^Foxp3^+^ Treg cells in the spleen was determined by flow cytometry. As shown in **Figure 4A**, the percentage of CD4^+^CD25^+^/CD4^+^T was 1.33% in rats in the CON group, compared with 0.89% in rats in the MOD group. Moreover, compared to rats in the MOD group, the ratio of CD4^+^CD25^+^/CD4^+^T among WEAV treated rats (*p* < 0.001) was 6.88, 8.08, and 8.26%, respectively, compared with 1.92% in VOAV.L-treated rats (*p* < 0.05). Our results also demonstrated that VOAV and WEAV treatment markedly increased the percentage of CD4^+^CD25^+^Foxp3^+^ Treg cells. WEAV treatment (3.45, 3.41, and 4.37%) (*p* < 0.001) showed even better effects when compared to VOAV treatment (1.55, 1.39, and 1.85%) (**Figure 4B**). Thus, these findings indicated that the therapy of VOAV and WEAV on IBD may associated with activation of Treg cells.

### Microbial Community Analysis

**FIGURE 5:**
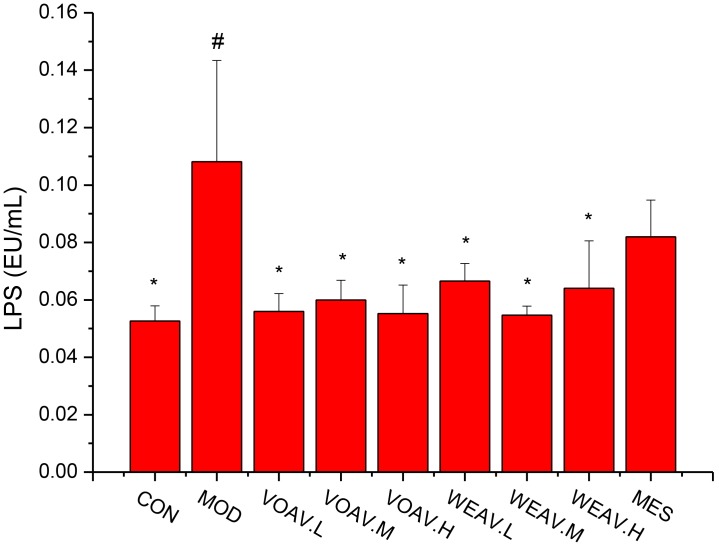
Effects of *A. villosum* administration on levels of lipopolysaccharide. Rats were induced by TNBS (100 mg/kg) in 50% ethanol solution. Various doses of VOAV and WEAV were orally administered. On the 9th day, hepatic portal vein blood samples of all rats were collected and determined by ELISA. Data are expressed as the mean ± SD (*n* = 6). The symbol “^∗^” indicates a significant difference compared with the model group, and “#” indicates a significant difference compared with the control group. ^∗^*p* < 0.05, ^∗∗^*p* < 0.01, ^∗∗∗^*p* < 0.001, ^#^*p* < 0.05, and ^###^*p* < 0.001. Relative abundance of microbial species in phylum level. Relative abundance of microbial species in Family level.

LPS, a cell-wall component of gram-negative pathogenic bacteria, is known to induce local or systemic non-special inflammatory lesions. The level of LPS in rats in the MOD group (0.11 ± 0.03 EU/mL) was significantly higher compared to that of rats in the CON group (0.05 ± 0.005 EU/mL) (*p* < 0.05) (**Figure 5**). All VOAV and WEAV treatments showed a significant effect on inhibiting the increase of LPS (*p* < 0.05), especially VOAV treatments.

The relation between the structure of intestinal bacterial flora and IBD has been widely investigated. A decrease of Firmicutes and an increase of Proteobacteria were observed in a IBD patient (Katsuyoshi and Takanori, 2015). Moreover, a low level of Actinobacteria has been observed in children with IBD (Sjoberg et al., 2017). Therefore, we investigated the effects of WEAV and VOAV treatment on gut microbiota.

**FIGURE 6:**
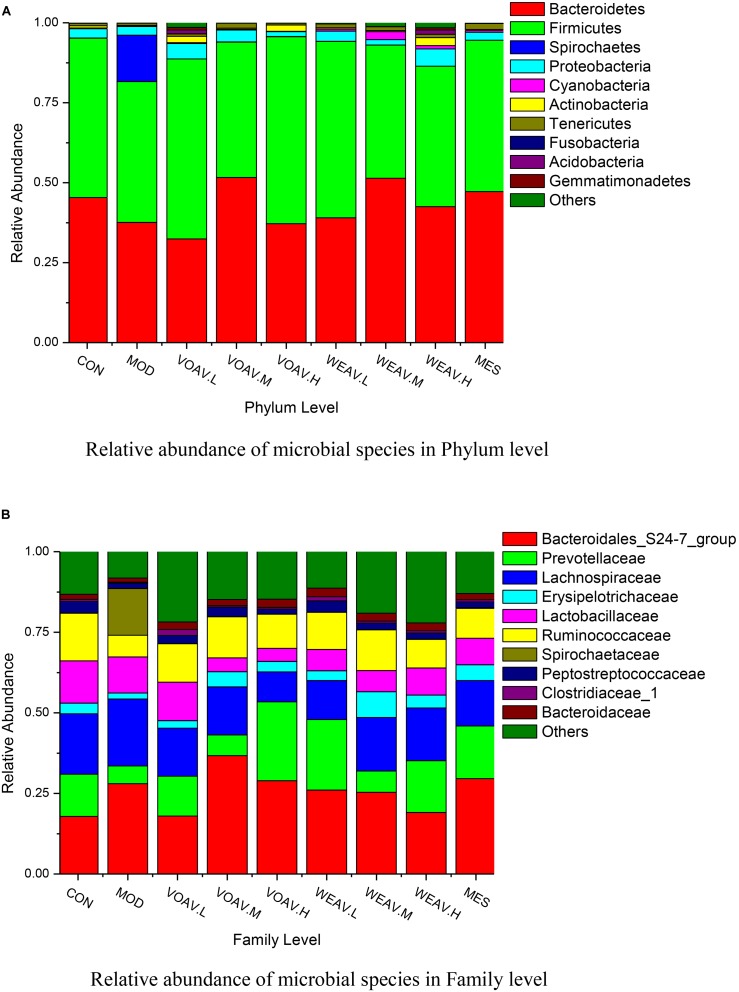
*Amomum villosum* and regulation of the balance of the intestinal microbial community. Three stool samples were randomly chosen, bar charts showing the relative abundance of the microbial community in phylum **(A)** and family level **(B)** in different stool samples.

Relative abundances in the level of phylum showed that (**Figure 6A**), dominant phylum across all samples included Firmicutes (41.6–58.5%), Bacteroidetes (32.4–51.6%), Proteobacteria (1.5–5.3%) and Actinobacteria (0.25–2.4%). In the MOD group, the sum of abundances of Firmicutes and Bacteroidetes decreased from 95.18 to 81.62%, while the abundance of Spirochaetes increased from 0.06 to 14.5%. In a study conducted by Helbling et al., one pediatric case was reported. The most prominent clinical manifestations of intestinal spirochetosis in children included abdominal pain, diarrhea, and rectal bleeding, which were similar to the characteristics of IBD (Rossana et al., 2012). In our study, we showed that treatment with VOAV (VOAV.H) and WEAV (WEAV.M) significantly increased the relative abundance of Firmicutes and Bacteroidetes, while a decrease in Proteobacteria was found. In addition, WEAV.M treatment showed a selective enrichment of Cyanobacteria (2.5%), while VOAV.H, VOAV.L, and WEAV.H treatment only affected Actinobacteria at the level of 2.0, 2.0, and 2.5%, respectively. These results were similar to the findings reported by Stearns et al. (2011).

At the family level, the abundance of Prevotellaceae, Ruminococcaceae, Erysipelotricnaceae, and Peptostrepto-coccaceae in rats in the MOD group was 50% lower compared to that of rats in CON group, while the abundance of Spirochaetaceae reached 14.5% (**Figure 6B**). Both VOAV and WEAV treatment had a significantly modulating effect on the above-mentioned bacteria. Among them, VOAV.H and WEAV.L increased the abundance of Prevotellaceae to 24.5 and 21.9%, respectively. The abundance of Ruminococcaceae was increased to about 12.7% in both VOAV.M- and WEAV.M-treated rats, which is similar to that of rats in the CON group.

### Influence on Species Highly Associated With Pathogenesis of IBD

**FIGURE 7:**
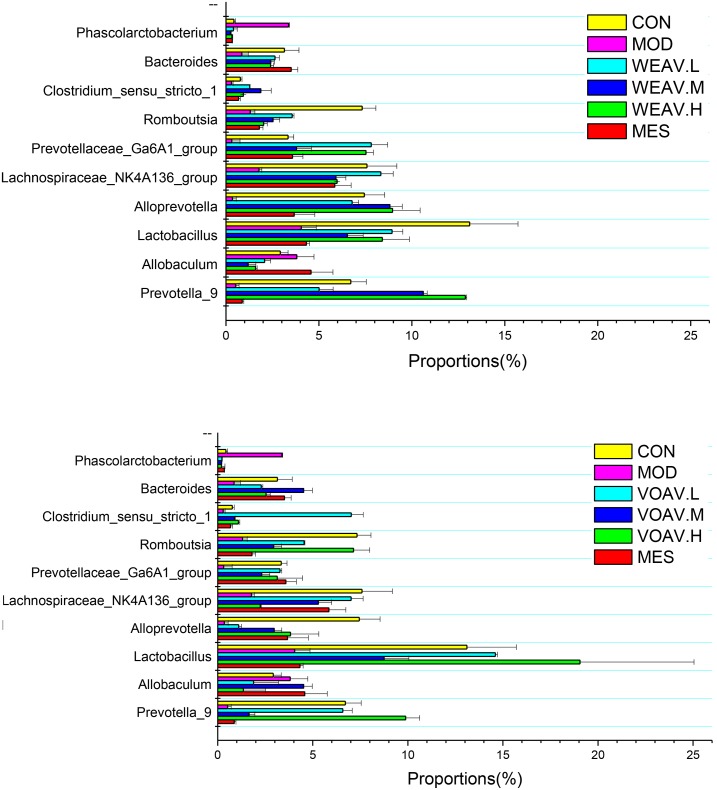
*Amomum villosum* and regulation of the intestinal microbial community. Three stool samples were randomly chosen, bar charts showing the proportions of the relative abundance of the microbial community at the genus level in different stool samples.

The results of the present study suggested that, low abundance of potentially anti-inflammatory microbes were closely related to the incidence of IBD (Katsuyoshi and Takanori, 2015; Sjoberg et al., 2017). At the genus level, the relative abundance of *Prevotella*, *Alloprevotella*, *Clostridium*, *Romboutsia*, and *Bacteroides* in the MOD group was low (0.78, 1.97, 0.32, 1.65, and 1.26%, respectively), compared with the CON group (6.71, 5.89, 0.77, 7.32, and 3.14%) (**Figure 7**). VOAV.H treatment affected *Romboutsia* and *Lactobacillus*, as their relative abundance increased to 7.14 and 19.1%. The abundance of *Prevotella* and *Clostridium* in VOAV.M-treated rats were 6.6 and 7.0%, respectively. WEAV.M treatment increased the abundance of *Prevotella* and *Alloprevotella* to 7.14 and 19.1%. In addition, WEAV.H treatment increased the abundance of Prevotella to 7.5%. The percentage of several genera including *Lactobacillus*, *Allobaculum*, and *Ruminococcaceae* was increased by VOAV.H, while *Blautia* levels were only increased by WEAV.H treatment. Enrichment of the short-chain fatty acid producers *Lactobacillus*, *Allobaculum*, *Ruminococcaceae*, and *Blautia* significantly protected pathogen-induced mucosal damage.

### Analysis of Cluster Heat Map

**FIGURE 8:**
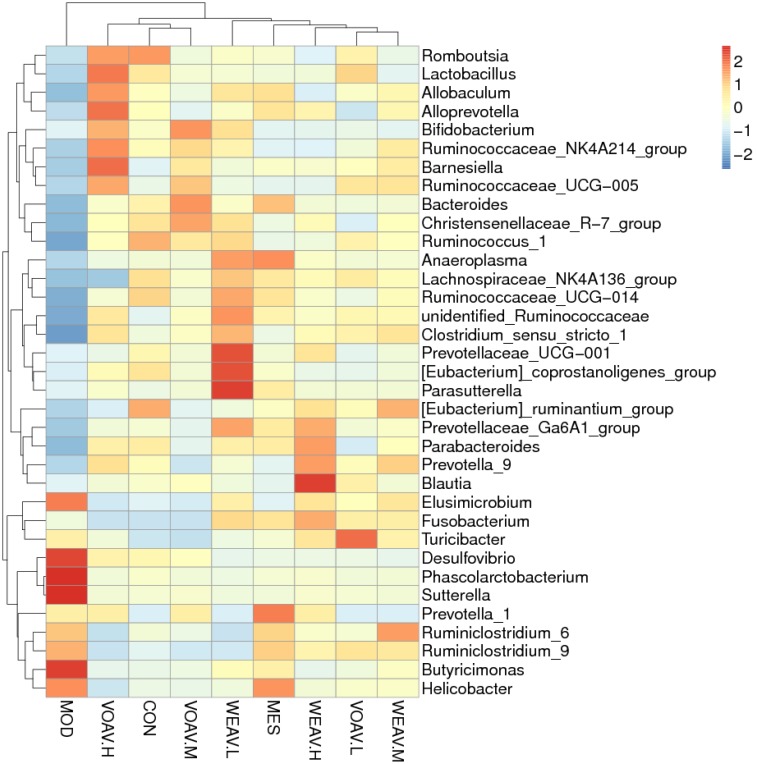
Cluster heat map analysis of the 35 most predictive operational taxonomic units (OTUs) belonging to the genus. Three stool samples were randomly chosen, Heat map represented by color ranging from blue, negative correlation (–2) to red, positive correlation (2). Abscissa axis represented sample information and vertical axis represents annotation information for species. The clustering tree on the left side indicates species clustering, on the top, sample clustering is shown. The values of the hot spot in the center represent *Z* values, which are standardized per species abundance per line.

According to the species annotations and the abundance information at the level of genus, heat maps of the 35 most predictive operational taxonomic units (OTUs) belonging to the genus were plotted, and cluster analysis was conducted (**Figure 8**). All experimental groups were divided into three categories: The MOD group was clustered into a single unit; VOAV.H, VOAV.M, and CON groups were clustered into one unit; and WEAV, VOAV.L, and MES groups were clustered in another group. Based on our findings, it may be concluded that VOAV treatment corrected intestinal flora disturbance to normal levels, and that WEAV-treated rats had similar intestinal flora regulating effect compared to rats in the MES-treated group.

### Correlation Between 35 Key Operational Taxonomic Units and Related Indexes

**FIGURE 9:**
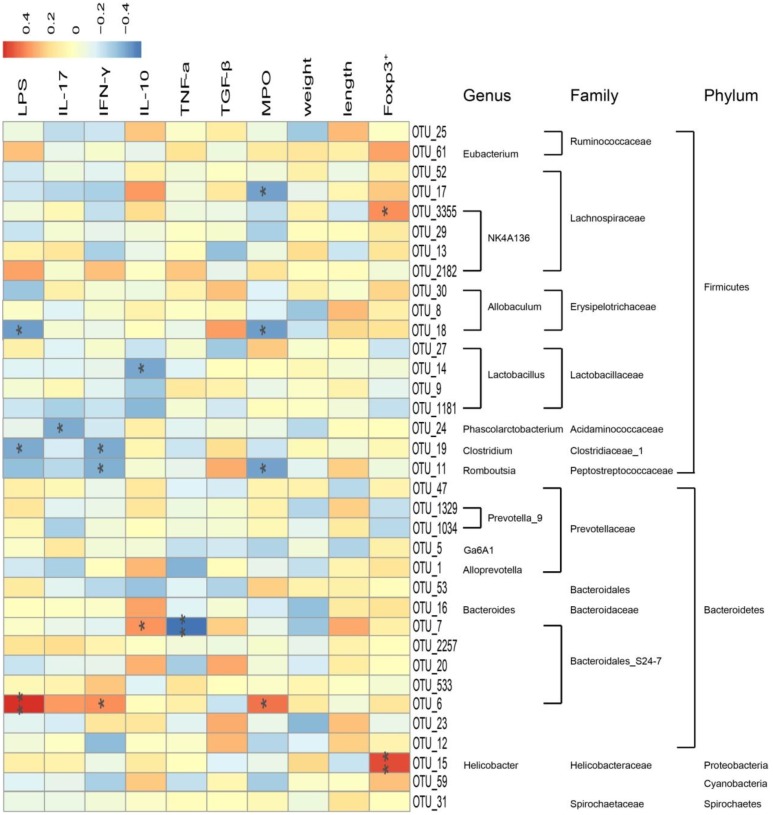
Correlation between 35 key operational taxonomic units (OTUs) and related indexes in rat. Data are expressed as the mean ± SD (*n* = 3). Correlation between the represented bacteria taxa information (genus, family, and phylum) of 35 key OTUs and related indexes in IBD rats. Pearson product moment correlation coefficients are represented by color, ranging from blue, negative correlation (–0.4) to red, positive correlation (0.4). Significant correlations after *P*-value adjustment are marked by ^∗^*p* < 0.05 and ^∗∗^*p* < 0.01.

Pearson correlation analysis was carried out based on clean data of the OTUs. As shown in **Figure 9**, a total of 35 key OTUs were identified and correlated to pharmacological indexes, such as LPS, IL-17, IFN-γ, IL-10, TNF-α, TGF-β, MPO, Foxp3^+^, colon weight, and colon length. Among them, Lachnospiraceae, Erysipelotricnaceae, Ruminococcaceae, Bacteroidaceae, and Prevotellaceae positively correlated with levels of IL-10, TGF-β, and Foxp3^+^. In addition, most Lachnospiraceae, Erysipelotricnaceae, Acidaminococcaceae, Clostridiaceae, and Peptostreptococcaceae negatively correlated with levels of LPS, IL-17, IFN-γ, and MPO activity. Nevertheless, most Lactobacillaceae negatively correlated with IL-10, whereas Helicobacteraceae positively correlated with Foxp3^+^. In addition, a portion of Bacteroidales_S24-7 negatively correlated with TNF-a levels and positively correlated with LPS. Among this family, the abundance of Erysipelotricnaceae, Ruminococcaceae, and Prevotellaceae was markedly increased after administration of *A. villosum*, indicating disturbance of intestinal flora combined with an abnormal immunoregulatory response in IBD development.

## Discussion

Trinitrobenzenesulfonic acid-induced IBD has been demonstrated as an ideal rat model for experimental studies on IBD given that it shares several clinical, histological, and immunological features with human CD disease. Ethanol (50%) damaged the gut mucosal barrier, whereas TNBS, acting as a hapten, induced a local mucosal immune response (Morris et al., 1989). The colon of TNBS-treated rats adhered with adjacent organs, causing a significant effect on colon weight and length (Li and Hauenstein, 2015). In the present study, we showed that VOAV and WEAV significantly improved general physiological features of IBD rats, such as body weight gain, food intake recovery, colon length, colon weight, and DAI scores. Treatment effects also included significantly ameliorated macroscopic and microscopic lesions of colons in TNBS-induced IBD rats.

Gut microbiota and an aberrant immune response are among the most accepted hypothesis of IBD pathogenesis. Accumulating evidence has indicated that T lymphocytes play a significant role in the immunological mechanisms of IBD. This was associated with an impaired immune response characterized by an imbalance between CD4^+^ Th cells and regulatory CD4^+^ T cells, which has been reported to regulate pro/anti-inflammatory cytokine production. Foxp3^+^ acts as a specific marker of Tregs and promotes the transition of naïve CD4^+^ T cells to responding CD4^+^ cells (Izcue et al., 2006). To a certain degree, Tregs inhibited Th1, Th2, and Th17, maintained intestinal homeostasis, and accelerated the repair of the tissue (Huang and Chen, 2016). Th1/Th17 cells that produced IFN-γ and IL-17 induced inflammation, whereas Th2/Treg cells that produced TGF-β and IL-10 expressed Foxp3 and exerted anti-inflammatory activity (Shevach, 2011).

A change in the cytokine profile from Th1 and Th17 to Th2 and Treg could ameliorate the progression of TNBS-induced colitis. Foxp3 dominates Treg formation and production of regulatory cytokines, such as TGF-β and IL-10. In our study, we showed that Th1/Th17 related cytokines, such as IFN-γ and IL-17 were markedly increased in rats in the MOD group, while the production of Th2/Treg associated cytokines TGF-β and IL-10 were reduces during the progression of colitis. Our results showed that the treatment with *A. villosum* could significantly upregulated cytokine levels of TGF-β and IL-10 and downregulated the level of IFN-γ. Using flow cytometry, we observed that the percentage of CD4^+^CD25^+^Foxp3^+^ Treg cells in the WEAV-treated IBD group were significantly higher than other groups.

LPS not only undermined the gut mucosal integrity but also promoted the intestinal lumen pathogenic agents into the bloodstream. In our study, we showed that treatment with *A. villosum* significantly inhibited LPS from entering the blood circulation. This inhibitory effect may be associated with the beneficial effects on the intestinal microflora equilibrium.

Considerable literature shows that more than 90% of all the bacterial species in the human gut belong to the *Bacteroidetes* and *Firmicutes* phyla, followed by lower proportions of *Proteobacteria* and *Actinobacteria* phyla (Eckburg et al., 2005). A decreased bacterial diversity and a greater bacterial instability have been found in patients suffering from IBD. Kang’s group reported that some bacteria belonging to the *Firmicutes* phylum including *Eubacterium rectale* of the Lachnospiraceae and *Ruminococcus albus*, *R. callidus*, *R. bromii*, and *F. prausnitzii* of the Ruminococcaceae were 5- to 10-fold more abundant in healthy persons compared to CD (Kang et al., 2010). A depletion of *Bacteroides* (Nemoto et al., 2012) and increases in bacteria belonging to the *Proteobacteria* (Frank et al., 2007) phyla have been observed in IBD patients. Our results were similar with the findings.

New insights indicated that symbiotic bacteria play an important role in calibrating the balance between intestinal flora and the immune response as colonization with segmented filamentous bacteria (SFB) and Clostridia induced Th17 and Treg cell differentiation in the intestine, respectively(Atarashi et al., 2013). Moreover, transferring feces from a healthy donor into the intestinal tract of a patient with IBD helps to recreate a favorable intestinal environment. Short-chain fatty acids (SCFA), as a main source energy in colonic epithelia, were very effective in upregulating the expression of Foxp3^+^, thereby protecting the mucosa from pathogen-induced damage, resulting in mitigating inflammation (Smith et al., 2013).

In our study, the Pearson correlation analysis confirmed that disturbance of the intestinal flora was closely related to the abnormal immunoregulation. Our results showed that Firmicutes and Bacteroidetes positively related to IL-10, TGF-β, and Foxp3^+^, whereas a negative correlation was found with IL-17, IFN-γ, MPO, and LPS. *Bacteroides*, *Prevotella*, and *Ruminococcus* that were largely accumulated in the gut microbiota of healthy human subjects had beneficial effects on the immune system (Gary et al., 2013). Administration of *A. villosum* significantly increased *Lactobacillus*, *Clostridiu*, *Ruminococcaceae*, *Lachnospiraceae*, *Bacteroides*, and *Prevotella*. Moreover, *A. villosum* decreased the abundance of Proteobacteria, such as *Desulfovibrio*, *Sutterella*, and *Helicobacter*. *A. villosum* also effectively increased SCFA-producing bacteria, thereby modulating the imbalance of intestinal flora.

Aqueous decoction is a traditional and widely used extraction method of traditional Chinese Medicine in clinic. Several investigations have shown that flavonoids, polysaccharide and organic acids are the main chemical composition of WEAV (Hu et al., 2005). Flavonoids are known to be effective antimicrobial substances against a wide array of microorganisms (Kumar and Pandey, 2013). Certain members of flavonoids including flavone and flavonol glycosides, isoflavones have shown the strongest antibacterial activity (Cushnie and Lamb, 2005), and significantly effect the function of the immune system and inflammatory cells (Tunon et al., 2009). Jeff’s group define innate immune responses induced by the polysaccharide component of Acai and have implications for the treatment of asthma and infectious disease (Holderness et al., 2011). Polysaccharides from Rhizome of *Polygonatum odoratum* showed antimicrobial activity against pathogenic bacteria *Staphylococcus aureus*, *Pseudomonas aeruginosa*, *Bacillus subtilis*, and *Escherichia coli* (Chen et al., 2014). Organic acid exhibited an inhibitory effect on the growth of a broad range of bacteria including growth of several important pathogens at high concentrations (Wu et al., 2017). The regulation effect of WEAV on the regulatory CD4^+^ cells and gut microbiota maybe related with these manifold physiological activators.

In our previous study, bornyl acetate (54.54%) was considered as the main active ingredient of the volatile oil and that it exerts its ant-inflammatory effect via modulation of p38 MAPK kinase and Caspase 3 expression, this could be an explanation for the observed effects in this study (Zhang et al., 2017). The results were the same as those reported in literatures (Zhang et al., 2011). Both kind of extracts (volatile and water extracts) showed different therapeutic effects. VOAV had an obvious inhibiting effects on inflammation, but the percentage of CD4^+^CD25^+^Foxp3^+^ Treg cells in the WEAV-treated IBD group were significantly higher than VOAV. High doses of VOAV and WEAV were able to increasing the diversity of dominant bacteria and inhibiting the activity of pathogenetic microbes.

Based on our studies and analysis of the relevant data, we speculated that the bioactivity of *A. villosum* was mediated by the synergistic effects of bornyl acetate, flavonoids, polysaccharide, and organic acids. This was concide with that Chinese medicinal herbs exert their pharmacological effects through a multi-component and multi-target way.

## Conclusion

In summary, *A. villosum* effectively relieved IBD by reversing the loss in body weight, and be decreasing the extent of the diarrhea and DAI scores. The expression level of pro-inflammatory cytokines, such as TNF-a, was significantly reduced by *A. villosum* therapy. *A. villosum* not only regulated Th1/Th2 and Th17/Treg-related cytokine production, but also promoted CD4^+^CD25^+^Foxp3^+^ Treg production. So we conclude that the therapeutic effect of *A. villosum* on IBD might be explained by its capability to induce Treg cells and rebalance CD4^+^ T cell subsets. Moreover, *A. villosum* may play a role in maintaining the internal environment by balancing the ratio between Firmicutes, Bacteroidetes, and Proteobacteria, thereby decreasing several opportunistic pathogens related to IBD and increasing SCFA-producing bacteria. In addition, endotoxemia in IBD rats was alleviated by *A. villosum*. Based on the above findings, *A. villosum* is effective in protecting against acute TNBS-induced colitis. Therefore, we conclude that the therapeutic of *A. villosum* on IBD probably connected with increasing the diversity of dominant bacteria, inhibiting the activity of pathogenetic microbes, maintaining the balance of intestinal microecology, promoting Treg differentiation and function, rebalancing CD4^+^ T cell subsets and alleviating intestinal inflammation. However, more profound researches about more detailed molecular mechanism are still needed in our future study.

## Author Contributions

ZC and WN conducted the study, analyzed the data, and prepared the paper. CY, TZ, and SL collected and interpreted the data. RZ, XM, and JY designed the study, provided the drugs and other facility, and critically corrected the manuscript.

## Conflict of Interest Statement

The authors declare that the research was conducted in the absence of any commercial or financial relationships that could be construed as a potential conflict of interest. The reviewer JO and handling Editor declared their shared affiliation.

## Abbreviations

Foxp3: forkhead box P3
IBD: inflammatory bowel disease
IFN-γ: interferon-γ
IL-10: interleukin 10
IL-17: interleukin 17
MES: mesalazin
MPO: myeloperoxidase
TGF-β: transforming growth factor-β
TNF-α: tumor necrosis factor-α
VOAV: volatile oil from *Amomum villosum* Lour.
WEAV: water extract from *Amomum villosum* Lour

## References

[B1] Atarashi K. Tanoue T. Oshima K. Suda W. Nagano Y. Nishikawa H. (2013). T_reg_ induction by a rationally selected mixture of Clostridia strains from the human microbiota. *Nature* 500 232–236. 10.1038/nature12331 23842501

[B2] Bell C. J. Gall D. G. Wallace J. L. (1995). Disruption of colonic electrolyte transport in experimental colitis. *Am. J. Physiol.* 268(4 Pt 1), G622–G630. 7733288 10.1152/ajpgi.1995.268.4.G622

[B3] Chen Y. Yin L. Zhang X. Wang Y. Chen Q. Jin C. (2014). Optimization of alkaline extraction and bioactivities of polysaccharides from rhizome of *Polygonatum odoratum*. *Biomed Res. Int.* 2014:504896. 10.1155/2014/504896 25093173 PMC4100354

[B4] Commission of Chinese Pharmacopoeia (2015). *Chinese Pharmacopoeia, China Medico-Pharmaceutical*, Vol. 1 Beijing: Science & Technology Publishing House.

[B5] Cushnie T. P. T. Lamb A. J. (2005). Antimicrobial activity of flavonoids. *Int. J. Antimicrob. Agents* 26 343–356. 16323269 10.1016/j.ijantimicag.2005.09.002 PMC7127073

[B6] Eckburg P. B. Bik E. M. Bernstein C. N. Purdom E. Dethlefsen L. Sargent M. (2005). Diversity of the human intestinal microbial flora. *Science* 308 1635–1638. 15831718 10.1126/science.1110591 PMC1395357

[B7] Fedorak R. N. Madsen K. L. (2004). Probiotics and the management of inflammatory bowel disease. *Inflamm. Bowel Dis.* 10 286–299. 15290926 10.1097/00054725-200405000-00018

[B8] Frank D. N. St Amand A. L. Feldman R. A. Boedeker E. C. Harpaz N. Pace N. R. (2007). Molecular-phylogenetic characterization of microbial community imbalances in human inflammatory bowel diseases. *Proc. Natl. Acad. Sci. U.S.A.* 104 13780–13785. 17699621 10.1073/pnas.0706625104 PMC1959459

[B9] Gary D. W. Frederic D. B. James D. L. (2013). Diet, the human gut microbiota, and IBD. *Anaerobe* 24 117–120. 23548695 10.1016/j.anaerobe.2013.03.011

[B10] Hang D. Y. Liu Y. (2015). The role of regulatory T cells in inflammatory bowel disease. *Int. J. Dig. Dis.* 35 42–45.

[B11] Hiroko N. K. Nobuhiko K. (2017). Host-microbial cross-talk in inflammatory bowel disease. *Immune Netw.* 17 1–12. 10.4110/in.2017.17.1.1 28261015 PMC5334117

[B12] Holderness J. Schepetkin I. A. Freedman B. Kirpotina L. N. Quinn M. T. Hedges J. F. (2011). Polysaccharides isolated from Açaí fruit induce innate immune responses. *PLoS One* 6:e17301. 10.1371/journal.pone.0017301 21386979 PMC3046208

[B13] Hu Y. L. Zhang Z. Y. Lin J. M. (2005). Advances in studies on chemical constituents and pharmacological activities of *Amomum villosum*. *J. Chin. Med. Mater.* 28 72–74.

[B14] Huang Q. Duan Z. Yang J. Ma X. Zhan R. Xu H. (2014). SNP typing for germplasm identification of *Amomum villosum* lour. Based on DNA Barcoding markers. *PLoS One* 9:e114940. 10.1371/journal.pone.0114940 25531885 PMC4274006

[B15] Huang Y. Chen Z. (2016). Inflammatory bowel disease related innate immunity and adaptive immunity. *Am. J. Transl. Res.* 8 2490–2497. 27398134 PMC4931145

[B16] Izcue A. Coombes J. L. Powrie F. (2006). Regulatory T cells suppress systemic and mucosal immune activation to control intestinal inflammation. *Immunol. Rev.* 212 256–271. 16903919 10.1111/j.0105-2896.2006.00423.x

[B17] Kang S. Denman S. E. Morrison M. Yu Z. Dore J. Leclerc M. (2010). Dysbiosis of fecal microbiota in Crohn’s disease patients as revealed by a custom phylogenetic microarray. *Inflamm. Bowel Dis.* 16 2034–2042. 10.1002/ibd.21319 20848492

[B18] Katsuyoshi M. Takanori K. (2015). The gut microbiota and inflammatory bowel disease. *Semin. Immunopathol.* 37 47–55. 10.1007/s00281-014-0454-4 25420450 PMC4281375

[B19] Kumar S. Pandey A. K. (2013). Chemistry and biological activities of flavonoids: an overview. *Sci. World J.* 2013:162750. 10.1155/2013/162750 24470791 PMC3891543

[B20] Li L. J. (2014). *Medical Microecology. Ver. 1*, Vol. 7 Beijing: People’s Medical Publishing House, 146–147.

[B21] Li Y. Hauenstein K. (2015). New imaging techniques in the diagnosis of inflammatory bowel diseases. *Viszeralmedizin* 31 227–234. 10.1159/000435864 26557830 PMC4608604

[B22] Morris G. P. Beck P. L. Herridge M. S. Depew W. T. Szewczuk M. R. Wallace J. L. (1989). Hepten-induced model of chronic inflammation and ulceration in the rat colon. *Gastroenterology* 96 795–803. 2914642

[B23] Murthy S. N. Cooper H. S. Shim H. Shah R. S. Ibrahim S. A. Sedergran D. J. (1993). Treatment of dextran sulfate sodium-induced murine colitis by intracolonic cyclosporin. *Dig. Dis. Sci.* 38 1722–1734. 8359087 10.1007/BF01303184

[B24] Nemoto H. Kataoka K. Ishikawa H. Ikata K. Arimochi H. Iwasaki T. (2012). Reduced diversity and imbalance of fecal microbiota in patients with ulcerative colitis. *Dig. Dis. Sci.* 57 2955–2964. 10.1007/s10620-012-2236-y 22623042

[B25] Neurath M. F. Fuss I. Kelsall B. L. Stüber E. Strober W. (1995). Antibodies to interleukin 12 abrogate established experimental colitis in mice. *J. Exp. Med.* 182 1281–1290. 7595199 10.1084/jem.182.5.1281 PMC2192205

[B26] Ng S. C. Lam Y. T. Tsoi K. K. Chan F. K. Sung J. J. Wu J. C. (2013). Systematic review: the efficacy of herbal therapy in inflammatory bowel disease. *Aliment. Pharmacol. Ther.* 38 854–863. 10.1111/apt.12464 23981095

[B27] Pagliari D. Gambassi G. Piccirillo C. A. Cianci R. (2017). The intricate link among gut “immunological niche,” microbiota, and xenobiotics in intestinal pathology. *Med. Inflamm.* 2017:8390595. 10.1155/2017/8390595 29118468 PMC5651127

[B28] Peterson D. A. Frank D. N. Pace N. R. Gordon J. I. (2008). Metagenomic approaches for defining the pathogenesis of inflammatory bowel diseases. *Cell Host Microbe* 3 417–427. 10.1016/j.chom.2008.05.001 18541218 PMC2872787

[B29] Randhawa P. K. Singh K. Singh N. Jaggi A. S. (2014). A review on chemical-induced inflammatory bowel disease models in rodents. *Korean J. Physiol. Pharmacol.* 18 279–288. 10.4196/kjpp.2014.18.4.279 25177159 PMC4146629

[B30] Rossana H. Maria-Chiara O. Bernard V. (2012). Intestinal spirochetosis mimicking inflammatory bowel disease in children. *BMC Pediatr.* 12:163. 10.1186/1471-2431-12-163 23066991 PMC3480841

[B31] Shafran I. Burgunder P. Wei D. Young H. E. Klein G. Burnett B. P. (2015). Management of inflammatory bowel disease with oral serum-derived bovine immunoglobulin. *Ther. Adv. Gastroenterol.* 8 331–339. 10.1177/1756283X15593693 26557889 PMC4622288

[B32] Shevach E. M. (2011). Biological functions of regulatory T cells. *Adv. Immunol.* 112 137–176. 10.1016/B978-0-12-387827-4.00004-8 22118408

[B33] Sjöberg F. Barkman C. Nookaew I. Östman S. Adlerberth I. Saalman R. (2017). Low-complexity microbiota in the duodenum of children with newly diagnosed ulcerative colitis. *PLoS One* 12:e0186178. 10.1371/journal.pone.0186178 29049404 PMC5648149

[B34] Smith P. M. Howitt M. R. Panikov N. Michaud M. Gallini C. A. Bohlooly-Y M. (2013). The microbial metabolites, short chain fatty acids, regulate colonic Treg cell homeostasis. *Science* 341 569–573. 10.1126/science.1241165 23828891 PMC3807819

[B35] Stearns J. C. Lynch M. D. Senadheera D. B. Tenenbaum H. C. Goldberg M. B. Cvitkovitch D. G. (2011). Bacterial biogeography of the human digestive tract. *Sci. Rep.* 1:170. 10.1038/srep00170 22355685 PMC3240969

[B36] Tuñón M. J. García-Mediavilla M. V. Sánchez-Campos S. González-Gallego J. (2009). Potential of flavonoids as anti-inflammatory agents: modulation of pro-inflammatory gene expression and signal transduction pathways. *Curr. Drug Metab.* 10 256–271. 19442088 10.2174/138920009787846369

[B37] Wu H. M. Wu L. K. Zhu Q. Wang J. Qin X. Xu J. (2017). The role of organic acids on microbial deterioration in the *Radix pseudostellariae* rhizosphere under continuous monoculture regimes. *Sci. Rep.* 7:3497. 10.1038/s41598-017-03793-8 28615734 PMC5471291

[B38] Yang M. Lin H. B. Gong S. Chen P. Y. Geng L. L. Zeng Y. M. (2014). Effect of *Astragalus* polysaccharides on expression of TNF-a, IL-1β and NFATc4 in a rat model of experimental colitis. *Cytokine* 70 81–86. 10.1016/j.cyto.2014.07.250 25132256

[B39] Ye L. N. Cao Q. Cheng J. F. (2013). Review of inflammatory bowel disease in China. *Sci. World J.* 2013:296470. 10.1155/2013/296470 PMC3848381 24348149

[B40] Ye Y. Pang Z. Chen W. Ju S. Zhou C. (2015). The epidemiology and risk factors of inflammatory bowel disease. *Int. J. Clin. Exp. Med.* 8 22529–22542. 26885239 PMC4730025

[B41] Yoshiyuki G. Yosuke K. Hiroshi K. (2015). The gut microbiota and inflammatory bowel disease. *Infect. Autoimmun.* 47 383–396.

[B42] Zhang S. Wang Z. Wang T. Lin J. (2011). Composition and antimicrobial activities of essential oil of fructus Amomi. *Nat. Prod. Res. Dev.* 23 464–472.

[B43] Zhang T. Lu S. H. Bi Q. Liang L. Wang Y. F. Yang X. X. (2017). Volatile oil from Amomi fructus attenuates 5-fluorouracil-induced intestinal mucositis. *Front. Pharmacol.* 8:786. 10.3389/fphar.2017.00786 29170638 PMC5684147

[B44] Zou Y. Li W. Y. Wan Z. Zhao B. He Z. W. Wu Z. G. (2015). Huangqin-tang ameliorates TNBS-induced colitis by regulating effector and regulatory CD4+T cells. *Biomed Res. Int.* 2015:102021. 10.1155/2015/102021 26347453 PMC4539427

